# Effect of Paper and Aluminum Bagging on Fruit Quality of Loquat (*Eriobotrya japonica* Lindl.)

**DOI:** 10.3390/plants10122704

**Published:** 2021-12-09

**Authors:** Cao Zhi, Muhammad Moaaz Ali, Junya Zhang, Meng Shi, Songfeng Ma, Faxing Chen

**Affiliations:** 1 School of Food and Bioengineering, Fujian Polytechnic Normal University, Fuqing 350300, China; caozhi@fpnu.edu.cn; 2 Fujian Universities and Colleges Engineering Research Center of Modern Facility Agriculture, Fuqing 350300, China; 3 College of Horticulture, Fujian Agriculture and Forestry University, Fuzhou 350002, China; muhammadmoaazali@yahoo.com (M.M.A.); zhangjy0413@gmail.com (J.Z.); 1190305013@fafu.edu.cn (M.S.); 3200330041@fafu.edu.cn (S.M.); 4 Xiamen Housing Group, Wanshun Cultural Industry Investment Development Co. Ltd., Xiamen 360000, China

**Keywords:** aluminum bagging, fruit quality, anthocyanin, sugar–acid ratio, carotenoids, fruit color

## Abstract

Bagging regulates the fruit microenvironment and improves the quality and market value of fruits. It is a safe and ecofriendly technique to protect fruits from insect/pest infestation and multiple biotic and abiotic stresses. In the current study, the influence of fruit bagging was evaluated on the development and quality of loquat fruits. Fruits from a healthy loquat orchard (Cv. Zaozhong No.6), located in Fujian, China, were enveloped in paper (T1), aluminum (T2), and aluminum–polyethylene bags (T3), while unbagged fruits were maintained as control (T0). In general, fruit bagging improved fruit quality in terms of fruit physiological and biochemical attributes and protected fruits from physical damage. In particular, aluminum–polyethylene bagging enhanced fruit weight, length, and width by 1.37-, 1.18-, and 1.13-fold, respectively. Loquat fruits bagged with paper bags exhibited the maximum soluble sugar and lowest titratable acid content. Fruits treated with paper and aluminum–ethylene bags showed twofold higher sugar–acid ratio as compared to control. Aluminum–polyethylene bagging caused 66.67%, 55.56%, and 33.33% reductions in skin burn, fruit rotting, and black spot of loquat. The fruits bagged in aluminum and aluminum–polyethylene did not show insect or bird damage, while unbagged fruits had 14.70% and 17.65% insect and bird damage, respectively. Overall, the results suggest that paper, aluminum, and aluminum–polyethylene bagging improved fruit health by 75%, 131%, and 144%, respectively, as compared to control. To delineate bagging type-dependent effects, principal component analysis was performed. Paper bagging was positively correlated with fruit firmness, rotting, soluble sugars, sugar–acid ratio, and proline content. Aluminum bagging was highly associated with improvements in titratable acids, cystine, and methionine. Aluminum–polyethylene bags were correlated with fruit weight, size, peel thickness, edible rate, and certain amino acids.

## 1. Introduction

Loquat (*Eriobotrya japonica* Lindl.) is an evergreen fruit tree originated from the People’s Republic of China [1,2]. It belongs to the family Rosaceae, subfamily Maloideae. It is a rich source of vitamin A, vitamin B6, potassium, magnesium, and dietary fiber [3]. It is a very beautiful orange-colored fruit with a mild sweet and sour taste [4]. It is most widely grown in Japan, Korea, India, Pakistan, and the south–central region of China. It is also grown as an ornamental shrub in California [5]. China is the leading producer and exporter of loquat and grows it on more than 100,000 hectares. The annual production of loquat in China reaches up to 380,000 tons [6]. More than 30 species of loquat are being grown in temperate and subtropical regions of Asia [7].

During growth and maturation, loquat fruits are susceptible to insect pests, birds, diseases, and mechanical damage, which reduce their commercial value [8]. Bagging, a physical protection technique commonly applied to many fruits, can not only improve fruit visual quality, by promoting fruit coloration and reducing the incidences of fruit cracking and russet, but also change the microenvironment of fruit development, which has multiple effects on the inner quality of fruits [8,9,10,11]. It is an effective method to decrease pesticide residues and increase the commercial value of the fruits [12]. Bagging was first utilized in Japan in the 20th century for pears and grapes, and it is now widely applied in Asian countries (Japan, China, and Korea), Australia, and the USA to protect fruits from the surrounding environment (mainly from pathogens, as well as stresses related to temperature, water /humidity, and air movement) with a physical barrier around the fruit [13]. In fact, bagging essentially consists of enclosing a young fruit in a food bag by capping the bag with a ribbon or a clamp on the fruit stalk [14]. Isolating the fruit from the external environment protects it during development from mechanical or biotic damages especially in regions where fruits are prone to attacks by fungi, bacteria, insects, and even birds [13].

Considering the importance of the bagging technique with respect to fruit quality, the impact of different bagging materials on the fruit quality of loquat was studied. It was hypothesized that loquat fruits bagged with paper (~50% light transmittance), aluminum (~0% light transmittance), and aluminum–polythene bags (with different light transmittance, i.e., ~0% and 100%, respectively) would show a significant difference compared to unbagged fruits in terms of fruit physical, physiological, and biochemical attributes.

## 2. Results

### 2.1. Physical Damage and Overall Health of Loquat Fruits

**Figure 1:**
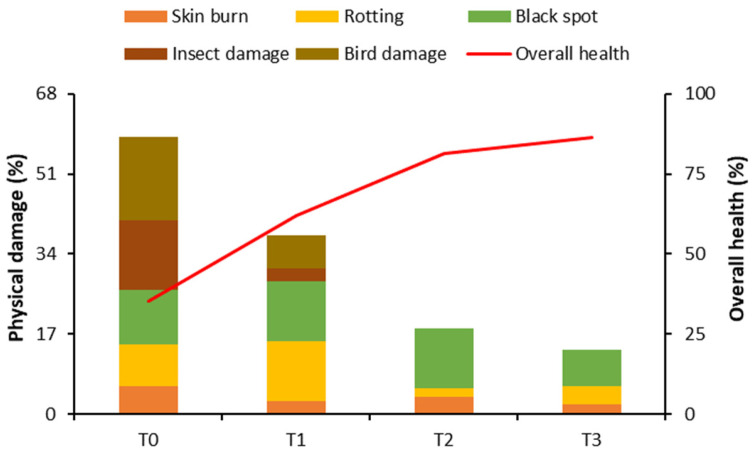
Effect of bagging treatments on physical damage in loquat fruits. T0—untreated fruits; T1 —single-layer paper bag; T2—aluminum foil bag; T3—aluminum–polyethylene bag.

Bagging treatments reduced fruit skin burn by up to 67% as compared to control. In the case of rotting, the maximum damage (12.68%) was recorded in the fruits treated with paper bags, while aluminum bags reduced rotting by 79.36%. Fewer black spots (7.84%) were observed in fruits bagged with aluminum–polyethylene bags (T3) as compared to other treatments. Interestingly, fruits treated with aluminum and aluminum–polyethylene bags remained protected against insect and bird damage, while fruits enveloped in paper bags showed 80.81% and 60.11% less insect and bird damage as compared to control, respectively. Overall, bagging treatments improved fruit health by up to 60% as compared to control (Figure 1).

### 2.2. Fresh Fruit Weight, Length, Diameter, and Fruit Shape Index

**Figure 2:**
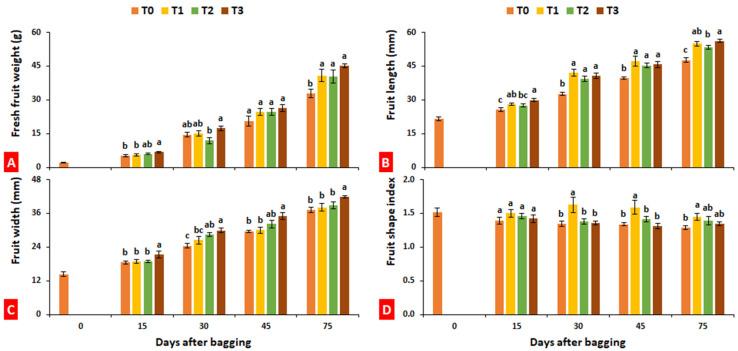
Effect of bagging treatments on fresh fruit weight (**A**), fruit length (**B**), fruit width (**C**), and fruit shape index (**D**) of loquat fruits. T0—untreated fruits; T1—single-layer paper bag; T2—aluminum foil bag; T3—aluminum–polyethylene bag. Different letters indicate significant differences (*p* ≤ 0.05) among bagging treatments on each day, according to Fisher’s least significant difference (LSD) technique.

Regardless of treatments, fresh fruit weight and fruit size increased with an increase in fruit maturity. After 45 days of bagging, fruit weight increased due to bagging treatment, regardless of bagging type, as compared to control. Aluminum–polyethylene bagging (T3) enhanced fresh fruit weight by 37.36% as compared to control (Figure 2A).

After 30 days of bagging, fruit weight increased due to bagging treatment, regardless of bagging type, as compared to control. An exception was observed in fruits bagged with aluminum 75 days after bagging when fruits exhibited a lower length than those treated with paper and aluminum–polyethylene bags. The maximum fruit length (56.14 mm) was exhibited by the fruited treated with aluminum–polyethylene bags (T3) after 75 days of bagging (Figure 2B).

Similarly, bagging significantly (*p* ≤ 0.05) improved the fruit width of the loquat. The maximum fruit width (41.85 mm) was exhibited by the fruited treated with aluminum–polyethylene bags (T3) after 75 days of bagging, which was 12.54% more than untreated fruits (Figure 2C). Paper bags significantly improved the fruit shape index of loquat, indicating its role in producing lengthy fruits as compared to other treatments (Figure 2D).

### 2.3. Fruit Firmness, Peel Thickness, Edible Rate, and Water Content

**Figure 3:**
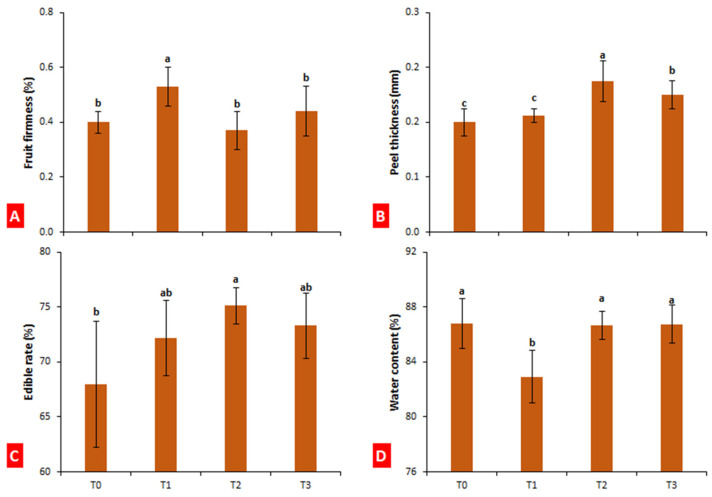
Effect of bagging treatments on fruit firmness (**A**), peel thickness (**B**), edible rate (**C**), and water content (**D**) of loquat fruits. T0—untreated fruits; T1—single-layer paper bag; T2—aluminum foil bag; T3—aluminum–polyethylene bag. Different letters indicate significant differences (*p* ≤ 0.05) among bagging treatments, according to Fisher’s least significant difference (LSD) technique.

Fruit firmness, peel thickness, edible rate, and water content were calculated at the last harvest of loquat fruits (75 days after bagging). Paper bags significantly (*p* ≤ 0.05) improved fruit firmness as compared to control (32.5% increase), while fruits enveloped in aluminum and aluminum–polyethylene bags did not show a significant difference compared to control (Figure 3A). Conversely, aluminum and aluminum–polyethylene bagging increased the peel thickness of loquat by 37.5% and 25%, respectively (Figure 3B). The fruits treated with aluminum bags significantly improved the edible rate of loquat as compared to control (10.55% increase), while fruits enveloped in aluminum and aluminum–polyethylene bags did not show a significant difference in edible rate as compared to control (Figure 3C). The water content remained unchanged with bagging treatment, except when treated with paper bags. Paper bags reduced the water content of loquat fruits by 4.44% as compared to control (Figure 3D).

### 2.4. Soluble Solids, Total Titratable Acids, Sugar–Acid Ratio and Soluble Sugars

**Figure 4:**
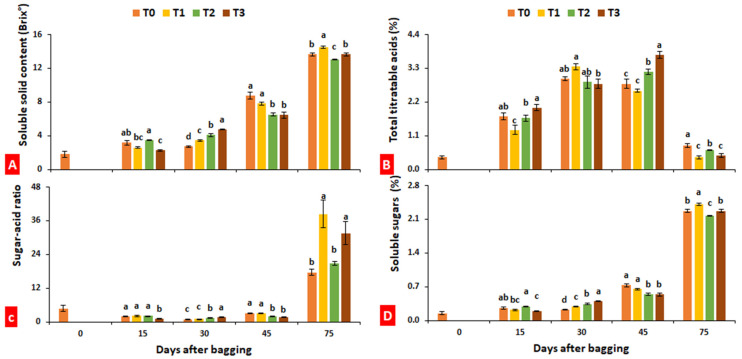
Effect of bagging treatments on soluble solid content (**A**), total titratable acids (**B**), sugar–acid ratio (**C**), and soluble sugars (**D**) of loquat fruits. T0—untreated fruits; T1—single-layer paper bag; T2—aluminum foil bag; T3—aluminum–polyethylene bag. Different letters indicate significant differences (*p* ≤ 0.05) among bagging treatments on each day, according to Fisher’s least significant difference (LSD) technique.

Soluble solid content (SSC) increased with an increase in fruit maturity, while total titratable acidity decreased with fruit maturity, showing reciprocal responses. There was a great variation in the change trend of soluble solids during fruit maturation within treatments. At final harvest (after 75 days of bagging), fruits enveloped in paper bags showed an increase in soluble solids by 6.15% as compared to control, while aluminum bagging reduced soluble solid content by 4.54% (Figure 4A). Conversely, total titratable acids increased until 45 days after bagging, before decreasing sharply. On the 75th day after bagging, overall titratable acids were found twofold reduced compared ot the 45th day after bagging, regardless of treatment. Furthemore, bagging treatment significantly (*p* ≤ 0.05) reduced the total titratable acidity of loquat fruits (Figure 4B). The sharp increase in sugar–acid ratio (Figure 4C) also indicates a sudden increase in total soluble solids and a decrease in titratable acids at the fruit ripening stage. Soluble sugars measured by the HPLC method also showed the same change trend as soluble solids (Figure 4D).

### 2.5. Fruit Color

Fruit peel color was measured by an automatic colorimeter in terms of the L*a*b* scale, where L = 100 stands for white and L = 0 stands for black, While “a+” and “a−” or “b+” and “b−” reflect for the color contrasts of red, green, yellow, and blue, respectively. The value of L* increased with increase in fruit maturity, indicating that loquat fruits become brighter with maturity. The highest values of L* were obtained in the fruits enveloped in aluminum and aluminum–polyethylene bags (59.67 and 59.72, respectively) at the fruit ripening stage. The negative values of a* and positive values of b* indicate that fruits were greenish-yellow at the ripening stage. The decreasing value of a* with fruit maturity indicates that fruit turned light green with fruit maturity. At the ripening stage, bagged fruits exhibited smaller a* than unbagged fruits. The minimum value of a* (65.71) was obtained in fruits bagged with aluminum–polyethylene bags. The increasing value of b* indicates that loquat fruits turned yellowish with fruit maturity. Aluminum–polyethylene bags significantly (*p* ≤ 0.05) increased b* in loquat fruits as compared to untreated fruits (Table 1).

### 2.6. Total Chlorophyll, Carotenoids, Anthocyanins, Flavonoids, and Phenolics

Chlorophyll data recorded after 15 days of bagging indicate that fruits bagged in paper bags had maximum chlorophyll (312.22 µg·g^−1^) compared to other treatments.

**Figure 5:**
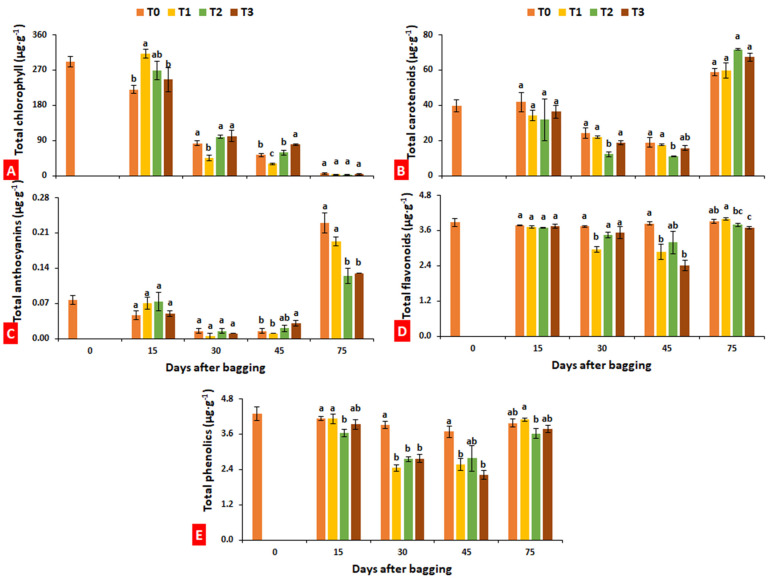
Effect of bagging treatments on total chlorophyll (**A**), carotenoids (**B**), anthocyanins (**C**), flavonoids (**D**), and total phenolics (**E**) of loquat fruits. T0—untreated fruits; T1—single-layer paper bag; T2—aluminum foil bag; T3—aluminum–polyethylene bag. Different letters indicate significant differences (*p* ≤ 0.05) among bagging treatments on each day, according to Fisher’s least significant difference (LSD) technique.

On the 30th and 45th days after bagging, a significant reduction was observed in the chlorophyll of the fruits of the same treatment. At full ripening stage (75 days after bagging), chlorophyll content was reduced to its minimum level, showing no significant (*p* ≤ 0.05) difference among treatments (Figure 5A). Total carotenoids and anthocyanins reduced with fruit maturity until 60 days after bagging, while a sharp increase was observed at last harvest (75 days after bagging) (Figure 5B,C). The results recorded at the ripening stage indicate that bagging treatments with aluminum and aluminum–polyethylene significantly reduced anthocyanins (Figure 5C). Bagged fruits showed slightly reduced flavonoids as compared to unbagged fruits, but the difference was comparable. The fruits treated with aluminum–polyethylene exhibited minimum flavonoids (3.7 µg·g^−1^) at the ripening stage among all treatments (Figure 5D). The phenolic content observed reduced with bagging treatments during fruit maturation, while, at full ripening stage, the fruits bagged with paper bags showed maximum phenolics (4.09 µg·g^−1^) among other treatments (Figure 5E).

### 2.7. Amino Acids

**Figure 6:**
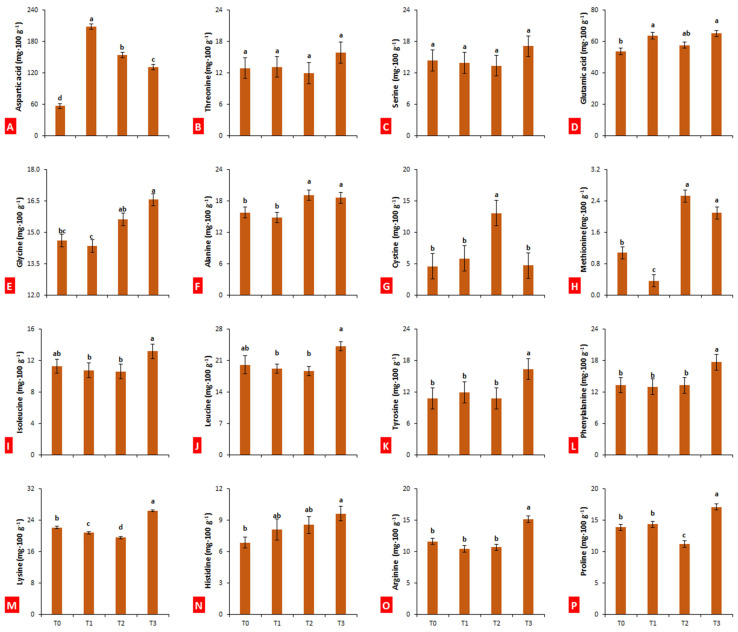
Effect of bagging treatments on aspartic acid (**A**), threonine (**B**), serine (**C**), glutamic acid (**D**), glycine (**E**), alanine (**F**), cystine (**G**), methionine (**H**), isoleucine (**I**), leucine (**J**), tyrosine (**K**), phenylalanine (**L**), lysine (**M**), histidine (**N**), arginine (**O**), and proline (**P**) of loquat fruits. T0—untreated fruits; T1—single-layer paper bag; T2—aluminum foil bag; T3—aluminum–polyethylene bag. Different letters indicate significant differences (*p* ≤ 0.05) among bagging treatments, according to Fisher’s least significant difference (LSD) technique.

Fruit bagging significantly (*p* ≤ 0.05) improved the contents of aspartic acid in the fruit pulp of loquat. The maximum value of aspartic acid (208.50 mg·100 g^−1^) was observed in the fruits enveloped in paper bags, while the minimum was observed in untreated fruits (56.92 mg·100 g^−1^) (Figure 6A). Threonine and serine remained unchanged with bagging treatments (Figure 6B,C). In the case of glutamic acid, fruits bagged in aluminum–polyethylene exhibited the maximum level (65.24 mg·100 g^−1^), followed by those bagged in paper bags (63.75 mg·100 g^−1^), whereas aluminum bagging had no effect on the content of glutamic acid (Figure 6D).

The maximum glycine (16.57 mg·100 g^−1^) was recorded in the fruits enveloped in aluminum–polyethylene (Figure 6E). In the case of alanine, cystine, and methionine, fruits bagged in aluminum bagged exhibited maximum values (19.15, 13.1, and 2.53 mg·100 g^−1^, respectively) (Figure 6F–H). Bagging treatments did not affect isoleucine and leucine contents in the fruit pulp of loquat, although aluminum–polyethylene bagging proved significantly better than paper and aluminum bagging (Figure 6I,J). In the case of tyrosine and phenylalanine, fruits treated with aluminum–polyethylene bags exhibited a maximum and significant level as compared to all other treatments (16.42 and 17.74 mg·100 g^−1^, respectively) (Figure 6K,L).

In the case of lysine, aluminum–polyethylene bagging significantly (*p* ≤ 0.05) improved, while paper and aluminum bags reduced the level (Figure 6M). Bagging with aluminum–polyethylene significantly (*p* ≤ 0.05) improved the histidine content of loquat fruits as compared to control (40.26% increase), whereas paper and aluminum bagging did not change histidine level as compared to control (Figure 6N). Similarly, in the case of arginine and proline, fruits treated with aluminum–polyethylene exhibited maximum contents (15.18 and 17.14 mg·100 g^−1^, respectively) (Figure 6O,P).

## 3. Discussion

The bagging technique is used specifically to enhance fruit appearance and quality, especially in Asia [14]. Bagging controls sunlight, temperature, humidity, evaporation, and mechanical damage to the fruits. It may also regulate harvesting time [15], and it can control pest attacks, minimizing the residues of pesticides [16,17,18]. In the present study, unbagged loquat fruits showed 14.70% and 17.65% insect and bird damage, respectively, while fruits bagged in aluminum and aluminum–polyethylene bags showed 0% pest infestation. Similarly, loquat fruits bagged in aluminum–polyethylene bags showed 66.67%, 55.56%, and 33.33% reductions in skin burn, fruit rotting, and black spot, respectively, indicating a positive role of the bagging technique in protecting the fruit from physical damage (Figure 1). Bagging is a very effective technique to modify the fruit microclimate [19]. The microclimate exhibited a positive influence on the structure of apple peels [20] and reduced the rotting in longan [19] and date palm [21], as well as fruit sunburn and cracking in pomegranate [22,23]. The bagging technique led to the production of more attractive fruits due to fewer blemishes and visible marks [13], particularly in apple [24,25,26,27], pear [17,28,29,30,31], peach fruits [11], mango [32,33,34,35,36], carambola [37], guava [38], litchi [10,39], loquat [8], and persimmon [40,41].

Bagging can increase fruit sugar and organic acid contents [11], two significant determinants of fruit organoleptic quality [42,43,44,45,46]. In the current study, fruits enveloped in paper bags showed a 6.15% increase in soluble sugars, while titratable acids were 50% reduced as compared to unbagged fruits (Figure 4). Sarker et al. [32] and Islam et al. [47] reported an increase in sugar content in bagged mango fruits, while Bently and Viveros [24] registered an improvement of fruit sweetness in Granny Smith apple. Conversely, Zhou et al. [48] reported a decrease in sugar content after bagging of Chinese white olives (*Canarium album* (Lour.) Raeusch.), as also found for apple [49] and date [50]. Huang et al. [51] stated that bagging had a nonsignificant effect on soluble sugars but decreased organic acids in pear fruits. Kim et al. [15] reported that peach fruits bagged with yellow paper showed an increase in total titratable acids due to low light, and white-colored bags determined an increase of soluble solid contents, chlorophyll, and anthocyanins.

In the present study, there was a nonsignificant difference between bagged and unbagged fruits in terms of chlorophyll and carotenoids, while anthocyanins were found significantly decreased in the fruits bagged with aluminum and aluminum–polyethylene. Aluminum–polyethylene bags significantly reduced the fruit flavonoids of loquat. Xu et al. [8] investigated the effects of different light-transmitting paper bags on fruits of two different cultivars of loquat (Baiyu and Ninghaibai); bagging materials included one-layer white paper bags ~50% light transmittance (Ta), and paper bags with a black inner layer and a gray outer layer with ~0% light transmittance (Tb). Fruit weight decreased, but fruit appearance improved with bagging, whereas total sugar content resulted higher in fruits subjected to Ta treatment compared to Tb and control. Both bagging materials reduced phenolics and flavonoids, with the lowest contents in Tb-treated fruits. Asrey et al. [52] reported that red cellulosic bags applied 60 days after flowering were successful in producing high-quality pomegranate fruits (characterized by high consumer acceptability) in terms of total anthocyanin and ascorbic acid content, whereas Yang et al. [19] observed that, in longan fruits, sugar content was not significantly affected by bag types but resulted in an increase in fruit size. In the current study, bagging improved fresh fruit weight and fruit size of loquat, regardless of bagging type (Figure 2). The intensity of the color tends to decrease in bagged fruits [14]; however, in the present study, fruits improved their color when treated with bagging, especially aluminum bagging (Table 1). Similarly, unbagging peach fruits 10 days before harvest restored a blush comparable to the control [53]. Zhou et al. [54] indicated that shortening the bagging period increases the anthocyanin level in peach peel but reduces peel brightness and chlorophyll content. Additionally, the effects of bagging on carotenoid content were studied in yellow-fleshed peach, for which the use of yellow–black double-layered bags significantly reduced the carotenoid level [55].

**Figure 7:**
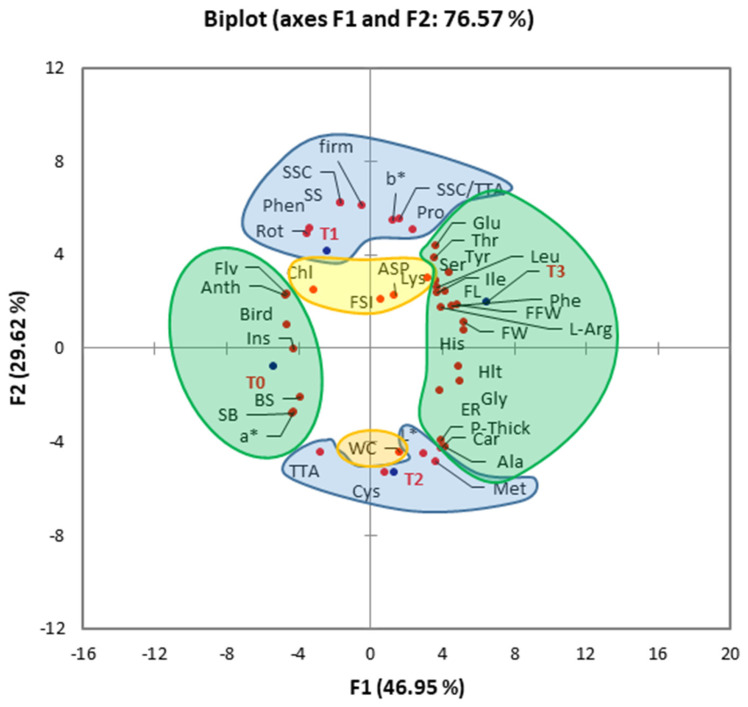
Principal component analysis among bagging treatments and various fruit quality attributes of loquat. Clustering of treatments and fruit quality attributes into groups (colored shapes) is based on their highest squared cosine values corresponding to the factor, F1 (green), F2 (blue), or F3 (yellow). Abbreviations: *FFW—*fresh fruit weight; *FL—*fruit length; *FW—*fruit width; *FSI—*fruit shape index; *Chl—*total chlorophyll content; *Car—*total carotenoids; *Anth—*total anthocyanins; *Flv—*total flavonoids; *Phen—*total phenolics; *SSC—*soluble solid content; *TTA—*total titratable acids; *SSC/TTA—*sugar–acid ratio; *SS—*soluble sugars; L*—fruit color (L*); a*—fruit color (a*); b*—fruit color (b*); *ASP—*aspartic acid; *Thr—*threonine; *Ser—*serine; *Glu—*glutamic acid; *Gly—*glycine; *Ala—*alanine; *Cys—*cystine; *Met—*methionine; *Ile—*isoleucine; *Leu—*leucine; *Tyr—*tyrosine; *Phe—*phenylalanine; *Lys—*lysine; *His—*histidine; *L-Arg—*arginine; *Pro—*proline; *SB—*skin burn; *Rot—*rotting; *BS*—black spot; *Ins—*insect damage; *Bird—*bird damage; *Hlt—*overall health; *firm—*fruit firmness; *P-Thick—*peel thickness; *ER—*edible rate; *WC—*water content.

Bagging can determine numerous changes in the physiology of the fruit and in the preservation of its characteristics, and its efficacy depends on its type and plant genetics [30]. Principal component analysis (PCA) was conducted to delineate the type-dependent effects of bagging on fruit quality of loquat (Figure 7). As a function of the highest squared cosine value corresponding to factors F1, F2, or F3, fruit quality attributes were clustered around T0, T1, T2, or T3 treatments. Factor F1, covering 46.94% variability in data (eigenvalue 19.717), showed clustering of fresh fruit weight, fruit length, fruit width, total carotenoids, threonine, serine, glutamic acid, glycine, alanine, isoleucine, leucine, tyrosine, phenylalanine, histidine, arginine, overall fruit health, fruit peel thickness, and edible rate with aluminum–polyethylene bagging (T3), suggesting its positive influence on these parameters. This cluster was located opposite to the control on the F1 axis, suggesting the strong positive influence of T3 on these parameters compared to control. Total anthocyanins, flavonoids, fruit color (a*), skin burn, black spot, insect damage, and bird damage showed a positive association with control (T0), indicating the negative correlation of bagging treatments with these parameters. The second factor, covering 29.68% variability in data (eigenvalue 12.441), showed clustering of total phenolics, soluble solids, sugar–acid ratio, soluble sugars, fruit peel color (b*), proline, fruit rotting, and fruit firmness with paper bagging (T1). However, the distribution of clusters in two distinct groups on either side of the F2 axis indicated that aluminum bagging had a positive correlation with total titratable acids, fruit color (L*), cystine, and methionine. The third factor of PCA, covering 23.43% variability in data (eigenvalue 9.842; not shown), showed clustering of fruit shape index, total chlorophyll content, aspartic acid, lysine, and fruit water content. Thus, the principal component analysis helped to delineate individual roles of bagging treatments in regulating various aspects of fruit quality attributes of loquat.

Overall, the results suggested that bagging promoted fruit growth and the quality of loquat. Paper (T1) and aluminum (T2) bagging was positively correlated with fruit firmness, sugars, acids, phenolics, proline, and fruit rotting, whereas aluminum–polyethylene (T3) bagging had a promotive impact on fruit weight, size, color, pigments, and amino acids.

## 4. Materials and Methods

### 4.1. Plant Material and Bagging Treatment

**Figure 8:**
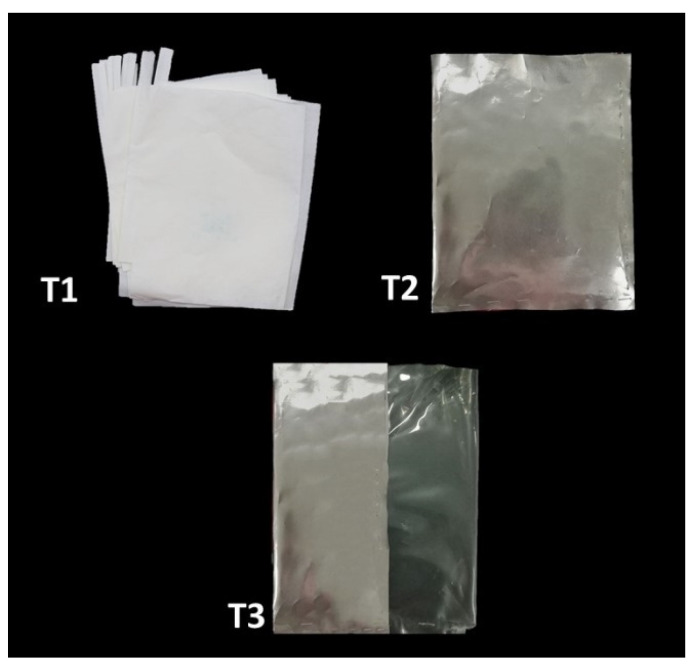
Bagging material used in the experiment. T1—single-layer paper bag; T2—aluminum foil bag; T3—aluminum–polyethylene bag.

Twenty young loquat trees (Cv. Zaozhong No.6) were selected from Minghuang Loquat Research Base located at Yunxiao, Fujian, China (23°57′13.5″ N, 117°20′36.0″ E). Loquat trees were 4–5.5 m tall with a canopy diameter of 4–5 m. The plantation distance among trees was approximately 6 × 6 m. Loquat trees were systematically pruned and thinned, and they were fertilized with NPK (15:15:15) at a concentration of 5 kg per plant per season, for the past three growing seasons. The study contained four treatments: control (no treatment) (T0), bagging with single-layer kraft paper with ~50% light transmittance (T1), bagging with aluminum foil with ~0% light transmittance (T2), and bagging with aluminum–polyethylene (one side of aluminum (~0% light transmittance) and another side of polyethylene (~100% light transmittance)) (T3) (Figure 8). Paper bags had good water resistance, as they were made of 80% long and 20% short fibers. Bagging material was autoclaved at 121 °C for 15 min before use. The fruits were bagged with paper and aluminum bags (25 cm × 36 cm) at the immature green stage. Each treatment was allocated to five loquat trees, considering each tree as a replicate. The measurement period was from November 2016 to February 2017. Before bagging, comprehensive pest control was carried out for the whole orchard, and then the orchard was routinely managed.

**Figure 9:**
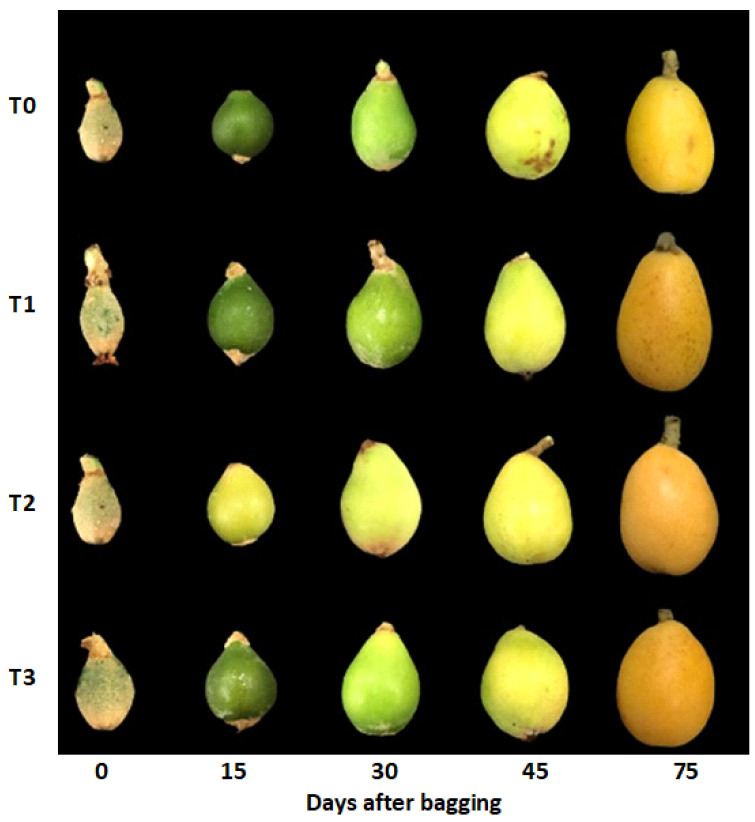
Loquat fruits harvested 0, 15, 30, 45, and 75 days after bagging. T0—untreated fruits; T1—single-layer paper bag; T2—aluminum foil bag; T3—aluminum–polyethylene bag.

Loquat fruits were bagged 70 days after flowering. Fruit quality data in terms of fruit weight, fruit size, soluble solid content, total titratable acidity, soluble sugars, and color pigments were collected after 0, 15, 30, 45, and 75 days of bagging. Fruits with the same growth and maturity were picked, and the samples were put into the sampling box and immediately brought back to the laboratory. Figure 9 shows fruits after bagging treatments sampled at different stages.

### 4.2. Measurement of Physical Damage and Overall Health of Loquat Fruits

At final harvesting (75 days after bagging), 60 ripe fruits with the same maturity from each treatment were randomly selected and classified into the fruits having skin burn, rotting, black spot, insect damage, bird damage, and healthy fruits. The percentage of damaged and healthy fruits was measured [16].

### 4.3. Fruit Fresh Weight, Length, Diameter, and Fruit Shape Index

Fruit fresh weight, length (from the maximum vertical point), and diameter (from the maximum horizontal point) were calculated by taking the average of 10 fruits from each treatment at each sampling stage. Fruit weight was measured with digital weighing balance (MJ-W176P, Panasonic, Osaka, Japan), whereas length and diameter were measured with digital Vernier calipers (DR-MV0100NG, Dongrun Imp. & Exp. Co., Ltd., Ningbo, China). Length was divided by the diameter of each fruit to calculate length-to-width ratio, hereafter called the fruit shape index.

### 4.4. Fruit Firmness, Peel Thickness, Edible Rate, and Water Content

Fruit firmness, peel thickness, edible rate, and water content were calculated by taking the average of 10 fruits from each treatment. Fruit firmness was measured with a digital fruit penetrometer (GY-4, Baoshishan, London, UK), whereas peel thickness was measured with digital Vernier calipers (DR-MV0100NG, Dongrun Imp. & Exp. Co., Ltd., Ningbo, China). To measure the edible rate of the fruit, peel and seeds were separated from pulp, and pulp weight percentage over total fruit was measured. Water content was measured by subtracting fruit dry weight from fruit fresh weight. To obtain dry mass, fresh fruits were dried in a hot air dehydrator (Ultimate 4000, Fowlers Vacola Australia Pvt. Ltd., Melbourne, Australia) until complete loss of moisture content in fruit tissues.

### 4.5. Soluble Solids, Total Titratable Acids, Sugar–Acid Ratio, and Soluble Sugars

Soluble solid contents were determined by placing a single drop of fruit juice on the circular screen of a handheld digital refractometer (Hybrid PAL-BXIACID F5, Atago, Tokyo, Japan). Total titratable acids were determined using the NaOH-based titrimetric method [56]. The sugar–acid ratio was calculated as a function of the soluble solid contents divided by total titratable acids within the same sample [43]. Soluble sugars were measured using high-performance liquid chromatography (HPLC) (Waters 2695 autosampler system, Waters Inc., Zellik, Belgium) as earlier described by Yu et al. [42].

### 4.6. Fruit Color

Fruit peel color was measured by taking the average of 10 fruits from each treatment. The color difference of three points on the fruit equator line was measured using an automatic colorimeter (PCE-CSM 2, PCE Instruments, Beijing, China), and the values of L*, a*, b*, c*, and h were recorded.

### 4.7. Total Chlorophyll, Carotenoids, Anthocyanins, Flavonoids, and Phenolics

To estimate chlorophyll pigments, 0.25 g of fresh fruit peel samples were chopped with scissors, transferred to a 50 mL tube containing 25 mL of 100% acetone, and kept in the dark at room temperature for 24 h. The sample was filtered, and absorbance was measured with a spectrophotometer (T60 U Spectrophotometer, PG Instruments Ltd., Leicestershire, UK). The chlorophyll and carotenoid contents were calculated using the following equations:

(1) Chl a=11.24 (A661.6)−2.04 (A644.8),

(2) Chl b=20.13 (A644.8)−4.19 (A661.6),

(3) Total Chlorophyll=7.05 (A661.6)+18.09 (A644.8),

(4) $$\mathrm{Total}\;\mathrm{carotenoids}=\frac{1000\left(A470\right)-1.9Chl\;a-63.14Chl\;b}{214},$$

where “A” denotes the sample used.

Total anthocyanins were extracted following the protocol earlier described by Kim and Lee [57]. Then, 0.2 g of plant material was added to 10 mL of 1% hydrochloric acid methanol solution and kept for 5 h. After centrifugation at 1000 rpm for 20 min, 10 mL of supernatant was used to measure the optical density (OD) of the sample at 530 nm and 560 nm. Equation (5) was used to calculate total anthocyanins.

(5) $$\mathrm{Total}\;\mathrm{anthocyanins}=\frac{\left(\mathrm{OD}530-0.25\times \mathrm{OD}650\right)\times \;\mathrm{volume}\;\mathrm{of}\;\mathrm{extraction}\;\mathrm{liquid}\;\left(\mathrm{mL}\right)}{4.62\times 104\times \;\mathrm{fresh}\;\mathrm{weight}\;\mathrm{of}\;\mathrm{passion}\;\mathrm{fruit}\;\left(g\right)}.$$

Extraction of total flavonoids was based on the optimization method of Vinatoru et al. [58] with some modifications. Accurately weighed 0.2 g of −80 °C frozen fruit peel was added to 8 mL of 60% ethanol. The solution was subjected to ultrasonic extraction for 40 min, cooling for 20 min, and centrifugation for 10 min (8000 rpm, 20°C). Then, 5 mL of supernatant was taken and diluted with distilled water to make the final volume of 10 mL. After that, a 2 mL aliquot was taken, and 3 mL of 60% ethanol and 0.3 mL of 5% NaNO_2_ were added. After shaking well for 6 min, 0.3 mL of 10% Al(NO_3_)_3_ was added. After shaking for 6 min, 4 mL of 4% NaOH was added. After shaking for 12 min, absorbance was measured at 510 nm. Total flavonoid content was calculated using a calibration curve (*Y* = 10.859, *X* − 0.0617, *R*^2^ = 0.999) of rutin standard (HPLC grade, ≥98% purity, Solarbio Life Sciences, Beijing, China).

To measure total phenolic content, 1 g of each sample (fruit peel) and gallic acid standard solution (20, 40, 60, 80, and 100 mg·L^−1^), 5 mL of Folin-Ciocâlteu reagent, and 4 mL of sodium carbonate (7% *w*/*v*) were added to a flask before shaking to mix the components completely. After keeping all samples in the dark for 30 min, absorbance was measured at 765 nm using a spectrophotometer (T60 U Spectrophotometer, PG Instruments Ltd., Leicestershire, UK). Reagent solution was used as a blank [59].

### 4.8. Determination of Amino Acids

Sample preparation was carried out according to the method of Hu et al. [60]. Briefly, the fruit samples were ground to a fine powder in the mortar with liquid nitrogen. Then, 3 g of powder was mixed with 2 mL of 60 mg·mL^−1^ 5-sulfosalicylic acid in a 10 mL centrifuge tube, before incubating in a water bath for 1 h at 37 °C. Then, 1 mL of 0.06 mol·mL^−1^ HCl and 1 mL of 10 mg·mL^−1^ EDTA-2Na were added and mixed well. The homogenate was then centrifuged at 13,500 rpm for 15 min by an Avanti J-26 XP centrifuge (Beckman Coulter, CA, USA). One milliliter of supernatant was mixed with 1 mL of pH 2.2 citric acid–citrate sodium buffer, followed by filtration with a 0.45 μm millipore filter membrane.

The amino acids were measured using an L-8800 amino-acid automatic analyzer (Hitachi High-Technologies, Tokyo, Japan), with a 20 μL filtrate injection, 855-350 ion exchange chromatographic column (4.6 mm × 60 mm, 3 μm), a column temperature of 134 °C, and a detection time of 125 min, monitored at the wavelengths of 570 nm and 440 nm.

### 4.9. Statistical Analysis

Collected data were subjected to ANOVA using statistical software “Statistix 8.1”. Treatment means were compared using Fisher’s least significant difference (LSD) method when *p* ≤ 0.05. Principle component analysis and correlation coefficient values were determined through the Pearson (*n*) technique using XLSTAT ver. 2019.

## 5. Conclusions

This study suggests that fruit bagging can be used to protect fruits from insect/pest infestation and increase the nutritional quality of loquats, two important factors contributing to economic profit and consumer health, respectively. Since fruit bagging with paper, aluminum, and aluminum–polyethylene bags differentially regulate distinct aspects of fruit quality, specific bagging materials may help to achieve the specific objectives of fruit production. Overall, aluminum–polyethylene bagging may be used as an effective preharvest application strategy to improve fruit health, increase yield, and improve the quality of loquat fruits. There is a need to investigate bagging-modulated molecular mechanisms regulating these fruit quality-related aspects. Since fruit bagging has been evaluated on only a few crops, there is also a need to expand and evaluate the performance of different bagging materials on horticultural crops.

## Figures and Tables

**Table 1:** Effect of bagging treatments on fruit color of loquat.

Days After Bagging	Treatment	L*	a*	b*
0	T0	32.40	−23.08	8.27
15	T0	28.74 b	−30.99 b	10.50 b
	T1	26.71 b	−26.78 b	8.69 b
	T2	39.70 a	−47.33 a	17.72 a
	T3	27.89 b	−26.02 b	8.55 b
30	T0	37.65 c	−47.35 a	16.77 c
	T1	39.97 cb	−49.70 ab	17.94 bc
	T2	48.10 a	−59.91 c	22.42 a
	T3	41.84 b	−55.60 bc	20.27 ab
45	T0	42.57 b	−44.69 a	19.09 c
	T1	46.26 b	−49.71 ab	20.00 bc
	T2	54.01 a	−60.54 bc	24.98 ab
	T3	53.25 a	−62.57 c	25.64 a
75	T0	58.63 ab	−46.29 a	26.31 ab
	T1	56.10 b	−61.99 b	26.37 ab
	T2	59.67 a	−57.76 ab	23.64 b
	T3	59.72 a	−65.71 b	27.83 a

T0—untreated fruits; T1—single-layer paper bag; T2—aluminum foil bag; T3—aluminum–polyethylene bag. Different letters indicate significant differences (*p* ≤ 0.05) among bagging treatments on each day, according to Fisher’s least significant difference (LSD) technique.
